# Retinal Microvascular Characteristics—Novel Risk Stratification in Cardiovascular Diseases

**DOI:** 10.3390/diagnostics15091073

**Published:** 2025-04-23

**Authors:** Alexandra Cristina Rusu, Klara Brînzaniuc, Grigore Tinica, Clément Germanese, Simona Irina Damian, Sofia Mihaela David, Raluca Ozana Chistol

**Affiliations:** 1 Doctoral School of Medicine and Pharmacy, Faculty of Medicine, University of Medicine, Pharmacy, Science, and Technology of Targu Mures, 540142 Targu Mures, Romania; alexandracristina.rusu@gmail.com; 2 Ophthalmology Center—Place de l’Etoile, Belair, 1371 Luxembourg, Luxembourg; 3 Faculty of Medicine, University of Medicine, Pharmacy, Science, and Technology of Targu Mures, 540142 Targu Mures, Romania; 4 Faculty of Medicine, “Grigore T. Popa” University of Medicine and Pharmacy, 700115 Iasi, Romania; grigore.tinica@umfiasi.ro (G.T.); sofia.david@umfiasi.ro (S.M.D.); raluca-ozana.chistol@umfiasi.ro (R.O.C.); 5 “Prof. Dr. George I.M. Georgescu” Cardiovascular Diseases Institute, 700503 Iasi, Romania; 6 Ophthalmology Department, Dijon University Hospital, 14 Rue Paul Gaffarel, 21079 Dijon, France; clement.germanese@chu-dijon.fr; 7 Institute of Forensic Medicine, 700455 Iasi, Romania

**Keywords:** retinal microvascularization, optical coherence tomography angiography, risk scores, artificial intelligence

## Abstract

**Background:** Cardiovascular diseases (CVDs) are responsible for 32.4% of all deaths across the European Union (EU), and several CVD risk scores have been developed, with variable results. Retinal microvascular changes have been proposed as potential biomarkers for cardiovascular risk, especially in coronary heart diseases (CHDs). This study aims to identify the retinal microvascular features associated with CHDs and evaluate their potential use in a CHD screening algorithm in conjunction with traditional risk factors. **Methods:** We performed a two-center cross-sectional study on 120 adult participants—36 patients previously diagnosed with severe CHDs and scheduled for coronary artery bypass graft surgery (CHD group) and 84 healthy controls. A brief medical history and a clinical profile were available for all cases. All patients benefited from optical coherence tomography angiography (OCTA), the use of which allowed several parameters to be quantified for the foveal avascular zone and superficial and deep capillary plexuses. We evaluated the precision of several classification models in identifying patients with CHDs based on traditional risk factors and OCTA characteristics: a conventional logistic regression model and four machine learning algorithms: k-Nearest Neighbors (k-NN), Naive Bayes, Support Vector Machine (SVM) and supervised logistic regression. **Results:** Conventional multiple logistic regression had a classification accuracy of 78.7% based on traditional risk factors and retinal microvascular features, while machine learning algorithms had higher accuracies: 81% for K-NN and supervised logistic regression, 85.71% for Naive Bayes and 86% for SVM. **Conclusions:** Novel risk scores developed using machine learning algorithms and based on traditional risk factors and retinal microvascular characteristics could improve the identification of patients with CHDs.

## 1. Introduction

According to the latest Eurostat reports, cardiovascular diseases (CVDs) are responsible for 32.4% of all deaths across the European Union (EU), with the highest mortality rates registered in Bulgaria (54.5%, 7.1 times more than France), followed closely by Romania (52%) [1]. Most CVD deaths are linked to coronary heart diseases (CHDs) (1194 deaths per million inhabitants) [2], for which prevention and early diagnosis remain key strategies for disease management.

Currently, several CVD risk scores based on known risk factors are in general use (Table 1) [3]. Regrettably, this increased number of scores is explained by their variable accuracy—for instance, the Framingham Risk Score (FRS) overestimates the risk by up to 104% and Pooled Cohort Equations by up to 118% in all individuals [4] while underestimating risk, especially in type 2 diabetes mellitus (T2DM) patients (FRS by 2.6% and SCORE by 1%) [5]. Such inaccurate estimations might lead to incorrect treatment plans in specific population groups.

Microvascular dysfunction is an important cause of CVD and can be quantified through minimally invasive (the index of microcirculatory resistance determined during coronary catheterization) and non-invasive methods such as fundus photography and OCTA (optic coherence tomography angiography) of the retina [6]. Changes in the retinal microvasculature have been reported in CVD patients and linked to disease pathophysiology, thus showing their promise as potential biomarkers for future cardiovascular events [7]. Some of these parameters can be determined on classic eye fundus images (central retinal arteriolar equivalent (CRAE), central retinal venular equivalent (CRVE), arteriolar-to-venular ratio (AVR), tortuosity, lacunarity, and fractal dimensions), while others can only be determined on OCTA (optical coherence tomography angiography) scans (vascular and perfusion density in the superficial and deep capillary plexus, fractal dimensions, foveal avascular zone area and perimeter, vessel tortuosity, vessel length and diameter index) [8,9]. Narrower CRAE, wider CRVE, lower AVR and fractal dimensions have already been associated with an increased risk of CHDs, while lower superficial vascular density is correlated with worse CHD outcomes [10]. To date, all risk scores in use have been developed using well-established statistical methods (logistic regression, area under the curve, discriminant analysis), and none of them include retinal microvascular parameters. Nowadays, a subset of AI technology, namely machine learning (ML), allows for the development of multiple classification and prediction algorithms that could improve cardiovascular risk stratification, such as Naive Bayes, Random Forest, Support Vector Machine (SVM) and k-Nearest Neighbors (k-NN).

This study aims to identify OCTA parameters significantly associated with CHDs and evaluate their potential use in a screening algorithm in conjunction with traditional risk factors. Moreover, the discriminative power of both conventional statistical methods and ML algorithms was assessed.

## 2. Materials and Methods

A two-center cross-sectional study was performed on 120 adult participants selected from the RASTA dataset of the Department of Ophthalmology at the University Hospital of Dijon (France) and the database of the Ophthalmology Center—Place de l’Etoile (Luxembourg). Of these, 36 patients diagnosed with severe CHDs based on coronary angiography and scheduled for coronary artery bypass graft surgery (CHD group) and 84 individuals with no significant cardiovascular pathology based on cardiological evaluation and cardiovascular history (control group) were assessed. From the RASTA dataset, we included patients from the RETINORM (75 normal volunteers) and MRCC (33 patients scheduled for coronary artery bypass grafting) subsets [11]. A total of 12 patients were added to these subsets, namely 3 patients with severe CHDs requiring surgical revascularization and 9 individuals with no significant cardiovascular pathology.

None of the study participants presented significant ophthalmologic disease interference with retinal microvascular features. The image acquisition technique used was described in the protocol established by Germanèse et al. [11].

A brief medical history and a clinical profile were available for all subjects. The following clinical and demographic characteristics were registered: age, sex, congestive heart failure, hypertension, diabetes mellitus, stroke history, vascular disease other than coronary, body mass index, CHA2DS2-VASc score, dyslipidemia and smoking at the time of evaluation.

All patients benefited from OCTA evaluation of the retina through the quantification of the following parameters: the foveal avascular zone (FAZ) perimeter, area and circularity index in the superficial capillary plexus; the vessel density in the superficial and deep capillary plexus (average, 3 × 3 mm and 6 × 6 mm macula centered on the fovea); the perfusion density in the superficial and deep capillary plexus (average, 3 × 3 mm and 6 × 6 mm macula centered on the fovea); the fractal dimensions of the average vessel density in the superficial and deep capillary plexus.

All retinal parameters except the fractal dimension were automatically quantified. The fractal dimension was measured on all images using Fractalyse 3.0 software (ThéMA Laboratoire, Université de Franche-Comté, Besançon, France) after prior binarization and skeletonization using ImageJ software version 1.54 (National Institutes of Health, Bethesda, MD, USA) [12].

SPSS (version 26, IBM) was used for statistical analysis, and *p*-values < 0.05 were considered significant. Descriptive statistics was used for summarizing baseline characteristics: mean ± standard deviation for continuous variables and counts/percentages for categorical variables. The intergroup comparison test (*t*-test or Mann–Whitney U test) was selected based on normality testing (Kolmogorov–Smirnov and Shapiro–Wilk tests). The chi-square test was used to evaluate the association between categorical variables, the Pearson correlation was used to assess the association between continuous variables and univariable linear regression was used to assess the association of categorical variables with continuous variables. Factors proving statistical significance in the univariate analysis were included in a multiple regression model to estimate the likelihood of a patient being diagnosed with severe CHD.

The same likelihood was also estimated using 4 ML classification algorithms (k-NN, SVM, Naive Bayes and supervised logistic regression) implemented in the Python 3.10 programming language [13] on the Google Colaboratory platform [14]. K-NN is a non-parametric lazy learning algorithm that classifies new data points based on the majority class of their k-Nearest Neighbors in the training data (the best value of k was estimated at 9 with minimum error for the current dataset), while SVM aims to find the optimal hyperplane that maximizes the margin between different classes, and Naive Bayes calculates the probability of a data point belonging to a particular class based on the probabilities of its features. We compared the prediction accuracy between the multiple regression model and ML algorithms to identify the optimal classification method. The ML models were compared in terms of precision, recall and F1-score.

Data were obtained in accordance with the Declaration of Helsinki. The RASTA dataset was processed under the rules established by the Ethics Committee of the University Hospital of Dijon, and data collection at the Ophthalmology Center—Place de l’Etoile (Luxembourg) was approved by the institution’s Ethics Committee.

## 3. Results

The clinical and demographic profiles of individuals included in this study are presented in Table 2. CHD patients registered an increased prevalence of comorbidities and risk factors compared to the control group.

The OCTA profiles of the same individuals are detailed in Table 3. Significant differences between the control group and CHD patients were registered for FAZ and superficial capillary plexus measurements. FAZ was more irregular, and its perimeter and area registered higher values, while vessel density, perfusion and branching complexity were lower in the superficial capillary plexuses of CHD patients compared to normal controls. Deep vascular plexus vessel density and perfusion registered no significant differences between the two groups.

Multiple logistic regression was applied to identify factors influencing the likelihood of CHD diagnosis (Table 4).

Traditional risk factors such as male gender, diabetes mellitus and advanced age significantly increased the likelihood of a CHD diagnosis, with a 71.3% classification accuracy based on this model. OCTA characteristics such as decreased superficial plexus fractal dimension, superficial vascular density 3 mm and superficial perfusion density 3 × 3 mm also increased the likelihood of positive CHD diagnosis, with a 76% classification accuracy (Table 4).

Combining the two models (traditional risk factors and OCTA) (Table 4) increased the classification accuracy to 78.7%.

The k-NN algorithm with an estimated k value of 9 had a classification accuracy of 72.5% using only clinical and demographic variables. After the addition of the OCTA characteristics of the superficial capillary plexus (fractal dimension, vessel density and vessel perfusion), the classification accuracy increased to 80.95% (Table 5).

Compared to conventional statistical analysis, the machine learning k-NN algorithm had a superior classification accuracy when using both traditional risk factors and OCTA characteristics (81% vs. 78.7%).

On the same dataset including clinical, demographic and OCTA variables, Naive Bayes had an 85.71% classification accuracy, superior to that of k-NN (Table 6).

The Support Vector Machine classifier tested on the same dataset had an 86% classification accuracy, almost identical to that of Naive Bayes but with a lower F1-score (Table 7).

Supervised logistic regression implemented as an ML algorithm had a classification accuracy of 81%, superior to that of traditional logistic regression in SPSS and similar to that of k-NN, but inferior to that of the Naive Bayes and SVM algorithms (Table 8).

## 4. Discussion

With constant innovations in fundus cameras and OCT technology facilitating precise retinal microvascular measurement, interest in retinal vascular imaging has increased significantly in the last two decades. For example, vessel caliber, tortuosity, lacunarity, optimality, branching angle or fractal dimension can be easily extracted from fundus images using semi-automated software tools such as SIVA, MONA-REVA or IVAN [15]. In the case of OCTA, it is possible to quantify the retinal microvascular network at the capillary level in different plexuses (vascular density, vascular perfusion, flow index, foveal avascular zone, etc.). Moreover, several studies published over the last two decades associate certain microvascular characteristics determined on fundus and OCTA images with an increased cardiovascular risk (morbidity or mortality) [9]. Accordingly, ocular biomarkers of systemic diseases are now proposed and conceptualized under the novel term “oculomics” [11].

Meanwhile, recent developments regarding the application of AI in medical imaging are promising for screening, diagnosis and prognosis evaluation. AI is gradually penetrating many aspects of everyday life, extending its coverage to include ophthalmology and cardiovascular medicine.

In cardiovascular medicine, risk stratification is a potential application of AI because traditional prediction models such as the Framingham Risk Score and SCORE2 rely on multiple data points and may have limitations in certain ethnic groups and patients with low or intermediate risk profiles [16]. By using AI algorithms and integrating novel factors such as retinal microvascular parameters, the prognostic value of already-validated risk scores could be improved. We found a significant amelioration of CHD prediction accuracy when using ML algorithms compared to conventional statistical tests and when combining traditional risk factors with retinal microvascular features quantified via OCTA. In our study, the classification accuracy increased from 78.7% (traditional risk factors associated with OCTA characteristics) when using conventional statistical tests to 81% with the k-NN algorithm and supervised logistic regression, as well as 86% with the Naive Bayes and SVM algorithms. Of the four ML algorithms employed in this study, we consider Naive Bayes to be the optimal one, with 85.71% accuracy and the highest F1-score.

Several other algorithms have been employed for risk prediction and screening in CVDs based on retinal characteristics by previously published studies. Syed et al. used the EfficientNet-B2 network to predict 10-year cardiovascular disease risk based on retinal images in patients with type 2 diabetes without myocardial infarction or stroke prior to study entry [17]. The AI model improved the prediction performance of the Pooled Cohort Equation Risk Score (AUC 0.728). Germanese et al. assessed the accuracy of ML and DL (Deep Learning) algorithms in predicting the CHA2DS2-VASc neurocardiovascular score based on SS (swept source) OCTA retinal images of patients from the same open-source RASTA dataset as the current research. Using the EfficientNetV2-B3 DL model, the neurocardiovascular risk group was correctly predicted in 68% of cases with a mean absolute error (MAE) of approximately 0.697 [18].

Arnould et al. focused on quantitative retinal microvascular features obtained from fundus images using SIVA and Angioplex software (Carl Zeiss Meditec AG) and trained a supervised ML algorithm to predict age, history of diabetes and history of hypertension [19]. Another study published in 2023 investigated two ML models and found that 3 × 3 mm OCTA images could reliably predict the presence of hyperlipidemia, diabetes, hypertension and congestive heart failure [20]. Retinal vascular density was also used to identify patients with acute stroke using ML models [21].

Diaz-Pinto et al. used ML to assess the risk of acute myocardial infarction (AMI) using minimal information and fundus images [22]. Based on demographic information and images from 11,000 patients, their model estimated left ventricular (LV) mass, LV end-diastolic volume and the risk of acute coronary syndrome (ACS) with sensitivity and specificity rates exceeding 70%.

Moorfields Eye Hospital and the University College London Institute of Ophthalmology developed the RETFound model based on generative AI in 2023 and trained it on over 1.6 million fundus images and OCT scans. RETFound was then adapted to detect cardiovascular and systemic conditions. RETFound outperformed various other models in diagnosing and assessing the prognosis of cardiovascular diseases such as AMI, heart failure and ischemic stroke [23]. Another study developed and validated a nomogram based on OCTA, clinical and paraclinical variables able to predict CHDs in patients with suspected angina pectoris with good discriminatory power (AUCs of 0.942 and 0.897 in the training and validation sets, respectively) [24].

We also identified studies comparing the performances of various ML algorithms. Pal et al. used multi-layer perceptron (MLP) and k-NN to identify patients with cardiovascular diseases using data from the University of California Irvine database [25]. The reported accuracy rates were 82.47% for MLP and 86.41% for k-NN. Subsequently, in 2024, Suhatril et al. evaluated 10-year cardiovascular risk based on the Framingham Heart Study Community dataset using decision trees, Naive Bayes, k-NN, logistic regression, and CNNs (convolutional neural networks), with variable accuracy between 77 and 85%, precision between 70 and 84%, recall between 77 and 85%, and AUC between 0.58 and 0.72 [26]. In our study, k-NN registered a similar performance to that of supervised logistic regression (81% accuracy) but underperformed compared to Naive Bayes and SVM (86% accuracy). All ML algorithms outperformed conventional logistic regression, which involves univariable and multivariable analyses that must be performed step by step compared to ML algorithms implemented in Python code that can be re-run on several databases in a matter of minutes on virtual preconfigured machines such as those offered by Google Colaboratory [14].

Most research involves supervised and self-supervised ML algorithms, but unsupervised ML could also be applied to detect novel risk factors. Hu et al. used electronic-medical-record-trained ML models to develop a new model for detecting prevalent CVDs and obtained an 85.06% classification accuracy [27], close to what we obtained using supervised algorithms with traditional risk factors and OCTA parameters. Thus, unsupervised ML can support the development of a robust model for cardiovascular disease detection by identifying new risk factors.

Image captures and data derived from photos offer numerous advantages, making them potential biomarkers for large-scale cardiovascular disease screening and prognosis. Compared to coronary CT (computed tomography), for example, a fundus or OCTA examination is completely non-invasive, is risk-free, and can be performed with cheaper equipment occupying 1/100th the space of a CT machine, making it much more widely accessible. Risk assessment based on retinal biomarkers using AI tools could be a cost-effective strategy in academic research and daily clinical practice. Moreover, oculomics could be expanded to general screening programs outside clinical practice after comprehensive validation through multinational clinical trials. The first step in this direction would be a large-scale comparison of predictions based on existing models (Framingham Risk Score for Hard Coronary Heart Disease, PROCAM, CHA₂DS₂-VASc, SYNTAX, SCORE, etc.) with those involving oculomics and AI.

Although we have demonstrated the efficiency and accuracy of using ML algorithms in predicting cardiovascular risk, we believe that AI applications have several limitations, primarily stemming from the lack of strict methodological and ethical guidelines. Reproducibility and generalization should be the goals of every AI model used in medical practice. Currently, comparing results and developing large multicenter studies is difficult, especially in the case of OCTA imaging for the following reasons:

- Several OCTA systems have been released on the market, but images are not comparable because of the different image acquisition methods (swept source or spectral domain machines), laser wavelengths (varying between 840 and 1050 µm) and image reconstruction algorithms used; scan densities; and lateral and axial resolutions [28].
- Different image segmentation algorithms are employed by both OCTA machines and researchers. For example, we used ImageJ for image binarization and Fractalyse for computing fractal dimensions, while others have used the FracLac ImageJ plugin [29].
- No “gold standard” AI risk assessment algorithm exists, so various algorithms are employed, and their performances are not evaluated using standardized methods and are not based on datasets specific to a certain population (since model performance is conditioned by the number of cases, many authors resort to large composite databases derived from multiple populations). Validation on large external databases would be necessary prior to generalizing the results yielded by certain algorithms.

The ideal solution would be to have a single machine and a single segmentation algorithm to create large datasets to assess the performances of AI algorithms.

Ethical concerns must also be raised when integrating AI into clinical practice, with OCTA and fundus images being unique and containing biometric data potentially usable for biometric identification in the future. Moreover, AI usage raises further concerns involving data protection, informed consent and autonomy, as well as addressability gaps between countries and social groups, social interaction and real medical consultation. Even if we believe that ML holds real potential for improving diagnostic accuracy by offering novel data analysis tools, integrating AI into healthcare is difficult, especially in clinical specialties, as patients need human interaction and look for empathy and a compassionate environment when interacting with medical professionals [30].

## 5. Conclusions

The addition of retinal microvascular feature analysis to traditional risk factors could provide incremental value in certain patient groups with CHDs. Furthermore, ML algorithms show increased classification and discrimination accuracy compared to conventional statistical analysis.

ML algorithms could support the development of novel risk scores, while including retinal microvascular features alongside traditional risk factors in screening protocols opens metaphorical doors for further research into identifying newer and more accurate cardiovascular disease biomarkers. Although oculomics shows significant promise in a variety of medical fields, it remains a novelty, and extended multinational trials are necessary to accurately establish whether an AI-based approach outperforms traditional prediction models.

## Figures and Tables

**Table 1:** Cardiovascular risk scores [3].

Risk Score	Variables
Systematic Coronary Risk Evaluation (SCORE)	age, sex, SBP, TC, smoking status
Pooled Cohort Equations Calculator	age, sex, SBP, treatment for hypertension, TC, HDL-C, T2DM, smoking status
Framingham Risk Score (FRS)	age, sex, SBP, TC, T2DM, smoking status
Assign Risk Score	age, sex, SBP, TC, T2DM, smoking, social deprivation, family history of CVDs
QRISK3 Score	age, sex, SBP, TC/HDL-C ratio, T2DM, smoking status, ethnicity, social deprivation, BMI, family history of CHDs, treatment for hypertension, rheumatoid arthritis, atrial fibrillation, stage 4 or 5 chronic kidney disease, migraine, corticosteroid use, systemic lupus erythematosus, treatment with atypical antipsychotic medications, severe mental illness, erectile dysfunction, variability in blood pressure
Prospective Cardiovascular Münster Risk Score (PROCAM)	age, SBP, LDL-C, HDL-C, triglycerides, T2DM, family history of MI and smoking status
CUORE Risk Score	age, sex, SBP, TC, HDL-C, T2DM, treatment for hypertension, smoking status
Reynolds Risk Score	age, sex, SBP, TC, HDL-C, HbA1c if diabetic, smoking, hsCRP, parental history of MI

SBP—systolic blood pressure; TC—total cholesterol; HDL-C—high-density-lipoprotein cholesterol; LDL-C—low-density-lipoprotein cholesterol; T2DM—type 2 diabetes mellitus; CVDs—cardiovascular diseases; CHDs—coronary heart diseases; BMI—body mass index; MI—myocardial infarction; HbA1c—hemoglobin A1C; hsCRP—high-sensitivity C-reactive protein.

**Table 2:** Clinical and demographic profiles.

Variable	Control Group (84)	CHD Group (36)	*p*
Age (years) (mean ± SD)	54.52 ± 19.82	68.77 ± 9.92	0.012
Male (No., %)	45 (53.57%)	27 (75%)	0.028
Smoking (No., %)	12 (14.29%)	3 (8.33%)	n.s.
Diabetes mellitus (No., %)	5 (5.95%)	12 (33.33%)	<0.001
Congestive heart failure (No., %)	0%	8 (22.22%)	-
Stroke (No., %)	0%	2 (5.56%)	-
Chronic kidney disease (No., %)	2 (2.38%)	4 (11.11%)	0.044
Arterial hypertension (No., %)	13 (15.47%)	19 (52.78%)	0.0218
Dyslipidemia (No., %)	9 (10.71%)	18 (50%)	<0.001
CHA₂DS₂-VASc score (high risk) (No., %)	5 (5.95%)	13 (36.11%)	<0.001
Obesity (No., %)	10 (11.90%)	8 (22.22%)	n.s.
Vascular disease (No., %)	2 (2.38%)	23 (63.88%)	<0.001

SD—standard deviation; CHD—coronary heart disease; n.s.—not significant.

**Table 3:** The OCTA profiles of the two groups.

Variable	Group	Mean	Standard Deviation	*p*
FAZ perimeter (mm)	Control	2.2718	1.09829	0.003
	CHD	3.5688	6.0254	
FAZ circularity index	Control	0.7068	0.10596	0.047
	CHD	0.66871	0.16825	
FAZ area (mm^2^)	Control	0.3044	0.25496	0.008
	CHD	0.5612	1.29650	
Average superficial vascular density (mm^−1^)	Control	18.9107	2.55796	0.007
	CHD	18.1578	2.72017	
Superficial vascular density 3 mm (mm^−1^)	Control	18.0159	3.02361	0.043
	CHD	17.9371	1.57348	
Superficial vascular density 6 mm (mm^−1^)	Control	19.0300	2.60268	0.019
	CHD	18.7124	1.31628	
Average superficial perfusion density (mm^−2^)	Control	0.4176	0.03615	0.014
	CHD	0.3817	0.07121	
Superficial perfusion density 3 mm (mm^−2^)	Control	0.3799	0.05623	0.078
	CHD	0.3438	0.07964	
Superficial perfusion density 6 mm (mm^−2^)	Control	0.4156	0.03994	0.022
	CHD	0.3792	0.07472	
Average deep vascular density (mm^−1^)	Control	11.5449	4.26118	0.109
	CHD	11.4854	3.46850	
Deep vascular density 3 mm (mm^−1^)	Control	10.6803	4.01668	0.108
	CHD	10.4364	3.17422	
Deep vascular density 6 mm (mm^−1^)	Control	11.6731	4.33789	0.090
	CHD	11.7287	3.42562	
Average deep perfusion density (mm^−2^)	Control	0.2310	0.08707	0.763
	CHD	0.2178	0.08127	
Deep perfusion density 3 mm (mm^−2^)	Control	0.2144	0.08579	0.834
	CHD	0.1912	0.07996	
Deep perfusion density 6 mm (mm^−2^)	Control	0.2324	0.08840	0.823
	CHD	0.2187	0.08346	
Superficial fractal dimension	Control	1.7430	0.01448	0.033
	CHD	1.7297	0.01508	
Deep fractal dimension	Control	1.7519	0.01448	0.776
	CHD	1.7471	0.01139	

FAZ—fovea avascular zone; CHD—coronary heart disease.

**Table 4:** Logistic regression results.

Variable	B	S.E.	Wald	dfs	Sig.	Exp(B)
Traditional risk factors						
Gender	1.971	0.610	10.428	1	0.001	7.176
Age	0.076	0.024	9.912	1	0.002	1.079
Diabetes mellitus	1.709	0.706	5.864	1	0.015	0.181
OCTA characteristics						
Superficial fractal dimension	−81.962	21.397	14.673	1	0.000	0.003
Superficial vascular density 3 × 3 mm	−17.281	8.107	6.419	1	0.004	185.612
Superficial perfusion density 3 × 3 mm	−14.547	7.002	4.317	1	0.038	207.742
Traditional risk factors and OCTA characteristics						
Gender	1.929	0.639	9.122	1	0.003	6.884
Age	0.075	0.024	9.477	1	0.002	1.077
Diabetes mellitus	1.518	0.736	4.258	1	0.039	0.219
Superficial fractal dimension	−44.106	22.884	3.715	1	0.054	0.002
Superficial vascular density 3 × 3 mm	−8.517	7.121	2.478	1	0.014	92.724
Superficial perfusion density 3 − 3 mm	−4.648	4.106	1.281	1	0.048	104.353

B—unstandardized regression weight; S.E.—standard error; dfs—degrees of freedom; Sig.—significance; Exp(B)—ratio change in the likelihood of the event of interest for a one-unit change in the predictor.

**Table 5:** The classification performance of the k-NN algorithm including OCTA variables.

	Precision	Recall	F1-Score
CHD absent	0.82	0.93	0.88
CHD present	0.75	0.50	0.60
Accuracy			0.81
Macro average	0.79	0.72	0.74
Weighted average	0.80	0.81	0.80

**Table 6:** The classification performance of the Naive Bayes algorithm.

	Precision	Recall	F1-Score
CHD absent	0.93	0.87	0.90
CHD present	0.71	0.83	0.77
Accuracy			0.86
Macro average	0.82	0.85	0.83
Weighted average	0.87	0.86	0.86

**Table 7:** The classification performance of the SVM algorithm.

	Precision	Recall	F1-Score
CHD absent	0.88	0.93	0.90
CHD present	0.80	0.67	0.73
Accuracy			0.86
Macro average	0.84	0.80	0.82
Weighted average	0.85	0.86	0.85

**Table 8:** The classification performance of the supervised logistic regression algorithm.

	Precision	Recall	F1-Score
CHD absent	0.92	0.80	0.86
CHD present	0.62	0.83	0.71
Accuracy			0.81
Macro average	0.77	0.82	0.79
Weighted average	0.84	0.81	0.82

## Data Availability

Access to the RASTA dataset can be requested through the webpage https://rasta.u-bourgogne.fr/, accessed on 3 June 2024.
